# More than a Toxin: Protein Inventory of *Clostridium tetani* Toxoid Vaccines

**DOI:** 10.3390/proteomes7020015

**Published:** 2019-04-16

**Authors:** Jens Möller, Max Edmund Kraner, Andreas Burkovski

**Affiliations:** 1 Microbiology Division, Department of Biology, Friedrich-Alexander University Erlangen-Nuremberg, Staudtstr. 5, 91058 Erlangen, Germany; jens.moeller@fau.de; 2 Biochemistry Division, Department of Biology, Friedrich-Alexander University Erlangen-Nuremberg, Staudtstr. 5, 91058 Erlangen, Germany; max.kraner@fau.de

**Keywords:** *Clostridium tetani*, label-free quantification, neurotoxin, proteomics, tetanospasmin, vaccination

## Abstract

*Clostridium tetani* is the etiological agent of tetanus, a life-threatening bacterial infection. The most efficient protection strategy against tetanus is a vaccination with the *C. tetani* neurotoxin, which is inactivated by formaldehyde-crosslinking. Since we assumed that besides the tetanus toxin, other proteins of *C. tetani* may also be present in toxoid preparations, we analyzed commercially available vaccines from different countries in respect to their protein content using mass spectrometry. In total 991 proteins could be identified in all five analyzed vaccines, 206 proteins were common in all analyzed vaccines and 54 proteins from the 206 proteins were potential antigens. The additionally present proteins may contribute at least partially to protection against *C. tetani* infection by supporting the function of the vaccine against the devastating effects of the tetanus toxin indirectly. Two different label-free protein quantification methods were applied for an estimation of protein contents. Similar results were obtained with a Total Protein Approach (TPA)-based method and Protein Discoverer 2.2 software package based on the minora algorithm. Depending on the tetanus toxoid vaccine and the quantification method used, tetanus neurotoxin contributes between 14 and 76 % to the total *C. tetani* protein content and varying numbers of other *C. tetani* proteins were detected.

## 1. Introduction

Toxigenic strains of *Clostridium tetani* can cause tetanus, a life-threatening bacterial infection. Spores of this obligate anaerobic, saprophytic bacterium, which preferentially lives in warm and moist habitats, are present in environments all over the world and may enter the human body via contamination of wounds. These may be the result of injuries in case of adults or children or occur in case of newborns during birth when the umbilical cord is cut (neonatal tetanus). Under anaerobic conditions in dirt-contaminated wounds, poorly blood supplied or necrotic tissue, the spores may germinate and the vegetative cells can proliferate and produce exotoxin. Tetanospasmin, the extremely potent neurotoxin of *C. tetani*, blocks inhibitory neurotransmitters of the central nervous system, preventing relaxation of muscles and leading to muscle rigidity and spastic paralysis that are typical for tetanus [1]. Based on the frequent abundance of *C. clostridium* spores in the environment and a high fatality rate, which is almost 100% in case of lacking immune protection and intensive medical treatment, tetanus is a serious threat for human health. However, it can easily be prevented by vaccination with tetanus toxoid vaccine, which was already introduced in 1924, making it one of the oldest used vaccines (for review, see reference [2]).

Tetanus vaccine production is based on a *C. tetani* strain, designated as the “Harvard strain”, which was initially collected in the United States of America in the 1920s and subsequently distributed among scientists and vaccine producers worldwide [3]. In short, the bacteria are grown in liquid broth under anaerobic conditions, the toxin secreted by *C. tetani* is separated from bacteria and medium components using a filtration step and subsequently it is inactivated by cross-linking for four to six weeks or longer at 37 °C using 40% formaldehyde [4]. The resulting inactivated, crosslinked toxoid preparation may be used as a single antigen vaccine or in combination with other proteins to protect against diphtheria, pertussis and other vaccine-preventable diseases. Furthermore, tetanus toxoid is applied as a carrier protein in a number of conjugate vaccines. Applied either alone, in combination with other components as in diphtheria toxoid and tetanus toxoid (DT) vaccines or diphtheria, pertussis, and tetanus (DPT) vaccines or in co-administration with other vaccines, it is considered as safe and only mild local reactions may be expected [2,4].

Since it is well-known that bacteria may secrete different proteins into the medium during growth and also cell lysis may occur during cultivation in bio-reactors (e.g., [5,6,7,8,9]), we assumed that besides the tetanus toxin other proteins of *C. tetani* may also be present in toxoid preparations. To investigate this hypothesis, we used a recently developed protocol [10] to purify proteins from commercially available vaccines, reverse the formaldehyde cross-linking and analyze the protein inventory by tryptic digest and mass spectrometry.

## 2. Materials and Methods

### 2.1. Preparation of Vaccines Used in This Study

Commercially available diphtheria toxoid and tetanus toxoid (DT) vaccines used for proteome analyses are shown in Table 1. Since the protein content of the vaccine analyzed in this study was considered low, 10 vaccine doses (0.5 mL each) were pooled and precipitated by addition of 10% (*w*/*v*) trichloroacetic acid (TCA) and incubation at 4 °C for 16 h to get a concentrated sample for mass spectrometry analysis [11]. After incubating over-night at 4 °C the samples were centrifuged at 8000 x g for 30 min at 4 °C. The precipitated proteins were dried on ice and resuspended in rehydration buffer (2% sodium deoxycholate, 10 mM dithiothreitol (DTT), 50 mM Tris, pH 8.0). To reverse the formaldehyde cross-linking of the inactivated toxins, the samples were incubated for 20 min at 95 °C [12]. The protein amount one vaccine dose was determined using a spectrophotometer (NanoDrop LITE, Thermo Fisher Scientific, Bremen, Germany) at 280 nm.

### 2.2. Tryptic Digest and C18 Clean up

About 10 µg soluble proteins prepared from the vaccine samples (see above) were transferred to 10 kDa vivacon 500 membrane filters and the flow-through was discarded by centrifugation for 30 min at 12,000 × g. The tryptic digest of the prepared vaccines samples occurred within the modified Filter Aided Sample Preparation (FASP) protocol [11,17]. The proteins were reduced by addition of 200 µl of reduction buffer (25 mM DTT, 8 M urea, 50 mM triethylammonium bicarbonate buffer (TEAB)) for 30 min at 37 °C. Alkylation of sulfhydryl groups were carried out with 200 µl alkylation buffer (25 mM chloroacetamide (CAA), 8 M urea, 50 mM TEAB) for additional 30 min on a shaker at 600 rpm in the dark. The proteins were subsequently washed with 300 µl of 8 M urea in 50 mM TEAB followed by another washing step with 200 µL 6 M urea in 50 mM TEAB. Afterwards 0.5 µg mass spectrometry grade LysC endopeptidase was added onto the filter unit and incubated on a shaker at 37 °C and 600 rpm for 3 h, followed by a second digest with 1 µg trypsin and 250 µL dilution buffer (50 mM TEAB) to reach a final concentration of 1 M urea. The sample was incubated over-night at 37 °C at 600 rpm on a shaker. Peptides were then collected by centrifugation at 12,000 × g for 20 min. For acidification of the peptide solution 20 µL of 10% trifluoroacetic acid (TFA) was added to reach a final concentration of 0.5% TFA. To desalt the peptides a clean-up with C18 stage tips were performed. Prior to LC-MS/MS analysis, peptides were vacuum dried and resuspended in 0.1% trifluoroacetic acid (TFA) [8].

### 2.3. Mass Spectrometry

For mass spectrometric analyses peptides (250 or 2500 ng, respectively) were loaded onto a nanoflow Ultimate 3000 HPLC (Dionex, Sunnyvale, CA, USA). Separation was carried out using an EASY-Spray column (Thermo Fisher Scientific; C18 with 2 µm particle size, 50 cm × 75 µm) with a flow rate of 200 nl min^−1^ and increasing acetonitrile concentrations over 120 min. Method duration including equilibration and column wash was in total 160 min. All samples were analyzed on an Orbitrap Fusion mass spectrometer (Thermo Fisher Scientific, Waltham, MA, USA). The mass spectrometer was operating with 2000 V spray voltage, 275 °C transfer tube temperature, 300–2000 (*m*/*z*) scan range for the MS 1 detection in the Orbitrap, a maximum injection time of 50 ms, an automatic gain control (AGC) target of 400,000 and an Orbitrap resolution of 120.000. The most intense ions were selected for collision-induced dissociation with collision energy of 35%. For ion trap detection a maximum injection time of 250 ms and an AGC target of 100 were set [8,17]. Resulting raw data files were analyzed using the *Clostridium tetani* E88 database (Proteome Id: UP000001412) and the *C. diphtheriae* ATCC 700971/NCTC 13129/biovar gravis database (Proteome Id: UP000002198) in UniProt (www.uniprot/proteomes) and the Proteome Discoverer 1.4 program package (Thermo Fisher Scientific, Bremen, Germany). As described by Schäfer and co-workers [18] theoretical masses for peptides were generated by trypsin digestion with a maximum of two missed cleavages. Product ions were compared to the measured spectra using the following parameters: carbamidomethyl modification on cysteine was set as fixed and oxidation of methionine as dynamic modification. Mass tolerance was set to 10 ppm for survey scans and 0.6 Da for fragment mass measurements. For protein identification the thresholds were set on 1% false discovery rate (FDR).

### 2.4. Label-Free Quantitative Protein Analysis

For protein quantification, three samples from the German and Indian vaccine as well as three single vaccine doses from the Russian vaccine were prepared and analyzed by mass spectrometry. Based on the total protein approach (TPA) method [19,20] we used the peak area for protein quantification [21,22]. The total protein content was defined as the sum of peptide intensities integrated over the elution profile of each peptide and the amount of each identified protein was calculated as the ratio of their intensity to the sum of all intensities in the sample [23]. Only peaks ranged from 2 × 10^7^ up to 10^11^ where used for quantification [24]. In addition to the TPA-based quantification, the label-free quantification method based on the Minora algorithm [25] and included in the Protein Discoverer 2.2 program package (Thermo Fisher Scientific, Bremen, Germany) was applied.

### 2.5. Proteome Prediction of C. tetani

Cellular localization and the characteristics of *C. tetani* proteins identified in different samples of vaccines were extracted from the UniProt database. Proteins with unknown localization were analyzed by PSORTb v3.0.2 [26].

### 2.6. Prediction of Putative Antigens

A putative antigen function of proteins identified in the vaccine samples was determined using the VaxiJen database (http://www.ddg-pharmfac.net/vaxijen/VaxiJen/VaxiJen.html) with a threshold of 0.4 [27].

### 2.7. Data Availability Statement

Mass spectrometry results have been deposited to the ProteomeXchange Consortium (http://proteomecentral.proteomexchange.org) via the PRIDE partner repository [28]. Data are available via ProteomeXchange with identifier PXD009289.

## 3. Results

### 3.1. Mass Spectrometric Analysis of Tetanus Vaccines

#### 3.1.1. Identification of Proteins

**Figure 1:**
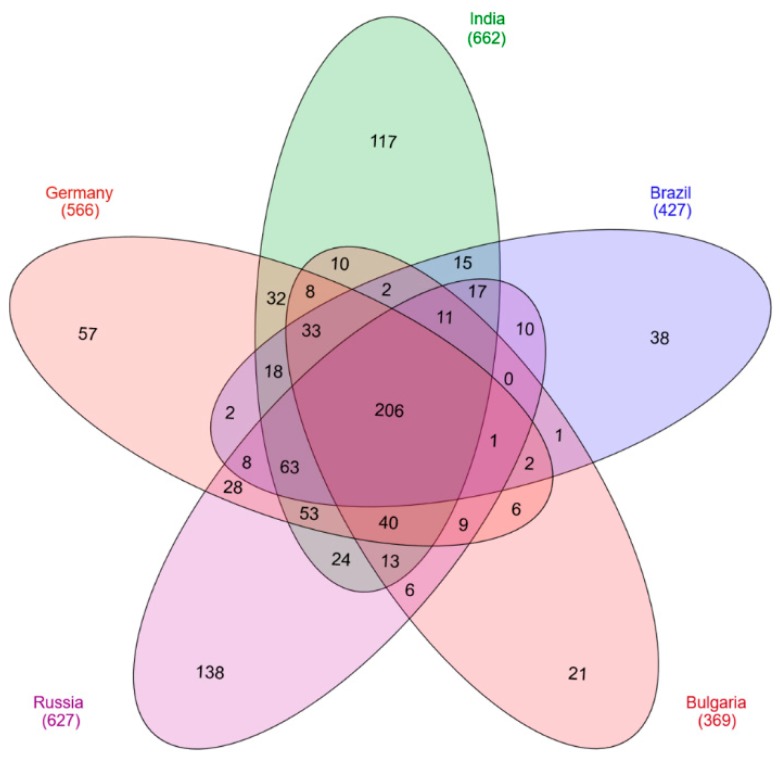
Venn diagram of identified proteins. 991 different proteins were found in the five vaccines analyzed with 206 distinct proteins observed in all vaccines.

For protein analysis, the processed vaccine samples were subjected to mass spectrometry. Identified peptides were analyzed with the Proteome Discoverer 1.4. For further investigations on protein localization and function the UniProt database was used. In total 991 *C. tetani* proteins were identified in all five analyzed vaccines (Table S1). Interestingly, the number of proteins identified in the different samples varied significantly. The highest numbers were observed for the vaccines from India and Russia with 662 and 627 proteins, respectively. The vaccine from Germany comprised 566 different proteins, 427 proteins were found in the vaccine sample from Brazil and 369 proteins in the Bulgarian vaccine. 206 unique proteins were present in all five analyzed vaccines (Figure 1).

#### 3.1.2. Protein Localization

**Figure 2:**
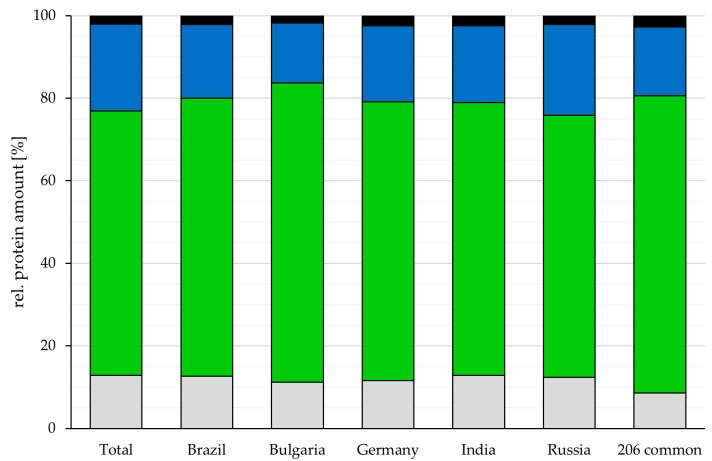
Localization of proteins identified in the analyzed vaccines. The proportion of proteins in respect to their predicted localization is shown for the vaccines from different countries and for the proteins common in all vaccines (grey: without predicted localization, green: cytoplasmic proteins, blue: membrane proteins and black: secreted proteins). For proteins with more than one annotated localization, each localization was added to the corresponding group.

While the number of proteins varied, the relative amount of secreted, membrane-located and cytoplasmic proteins as well as proteins with no predicted localization was rather similar among the different analyzed vaccines. Cytoplasmic proteins were 67.4 ± 3.3%, the major fraction in all vaccines (Germany: 67.6%, India: 66.1%, Brazil: 67.3%, Bulgaria: 72.4%, Russia: 63.5% and the common 206 proteins: 71.9%), followed by 18.3 ± 2.6% membrane proteins (Germany: 18.3%, India: 18.6%, Brazil: 17.9%, Bulgaria: 14.5%, Russia: 22.0% and the common 206 proteins: 16.7%) and 12.1% ± 0.7 proteins without predicted localization (Germany: 11.6%, India: 12.8%, Brazil: 12.6%, Bulgaria: 11.2%, Russia: 12.3% and the common 206 proteins: 8.6%), while secreted proteins were the smallest fraction with 2.2 ± 0.3% (Germany: 2.5%, India: 2.4%, Brazil: 2.2%, Bulgaria: 1.8%, Russia: 2.2% and the common proteins: 2.7%) (Figure 2). For 6.6 ± 1.0% of the proteins, two or more localizations were predicted (Germany: 6.3%, India: 5.7%, Brazil: 6.8%, Bulgaria: 5.6%, Russia: 8.1% and the common 206 proteins: 6.8%).

#### 3.1.3. Bioinformatic Analysis of the 54 Common Unique Proteins in All Analyzed Vaccines

**Figure 3:**
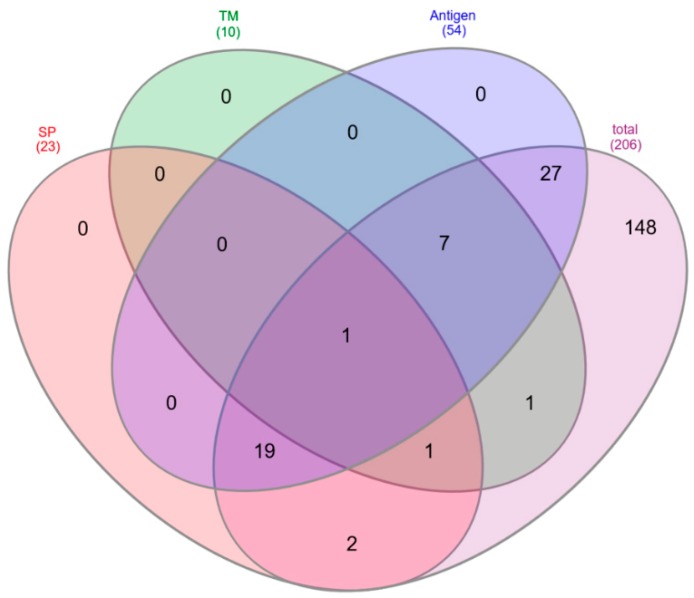
Properties of proteins common to all vaccines. Venn diagram of the 206 common proteins analyzed in respect to the presence of signal peptides (SP), transmembrane helices (TM) and predicted antigenic properties (Antigen).

To characterize the immunological potential of *C. tetani* proteins identified as parts of the tetanus toxoid vaccine, the proteins were analyzed in respect to the precence of signal peptides (SP) for protein translocation, transmembrane (TM) domains and their potential antigenic properties (Figure 3). From a total of 206 common proteins in all analyzed vaccines 23 proteins comprised a signal peptide (SP: 21, SP/TM: 2) and 10 proteins contained a transmembrane domain (TM: 8, TM/SP: 2), while 175 proteins comprised none of these motifs. Fifty-four proteins were predicted antigens based on a VaxiJen database screening (Table 2).

#### 3.1.4. Quantification of Proteins

The three vaccines with the highest number of different proteins were chosen for a more detailed characterization of the relative amounts of distinct proteins. In a first approach, DT vaccines were analyzed in respect to their protein content originating from *C. tetani* and *C. diphtheriae*. In all cases, the major part of the proteins of the DT vaccines analyzed were proteins from tetanus toxoid preparations, while *C. diphtheriae* proteins were present in much lower abundance, which corresponds to the different international units combined in the DT vaccines (see Table 1).

**Figure 4:**
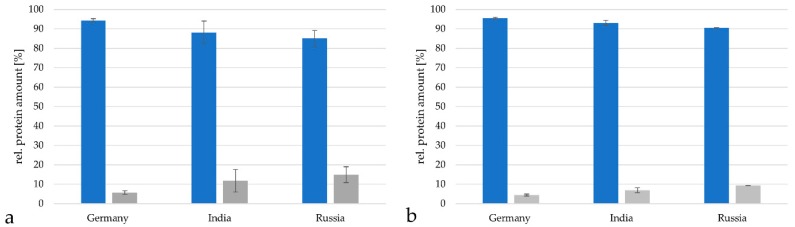
Relative protein amounts in DT vaccines. Comparison of relative protein amounts from *C. tetani* (blue) and *C. diphtheriae* (grey) in the vaccines from Germany, India and Russia. (**a**) Analysis by the TPA-based method using the area under the peak. Only proteins with peptides in in a range from 2 × 10^7^ to 10^11^ were considered for calculation. (**b**) Analysis with the Proteome discoverer 2.2 program package.

The vaccine from Germany contained 94.3 ± 0.9% proteins from *C. tetani*, the vaccine from India 88.1 ± 5.8% and the vaccine from Russia 85.1 ± 4.1% when analyzed with the TPA-based method described by [19,20,21,22] (Figure 4a). Proteins from *C. diphtheriae* reached 5.7 ± 0.88% in the German vaccine samples, 11.9 ± 5.8% in the Indian samples and 14.9 ± 0.1% in the Russian vaccine. When the Proteome Discoverer 2.2 program package based on the minora algorithm [25] was applied, a similar distribution was observed. In the German vaccine 95.5 ± 0.5% *C. tetani* and 4.5 ± 0.5% *C. diphtheriae* proteins were found, in the Indian vaccine 93.1 ± 1.3% *C. tetani* and 6.9 ± 1.3% *C. diphtheriae* proteins were observed and in the Russian vaccine 90.5 ± 0.1% *C. tetani* and 9.5 ± 0.1% *C. diphtheriae* proteins were detected (Figure 4b).

**Figure 5:**
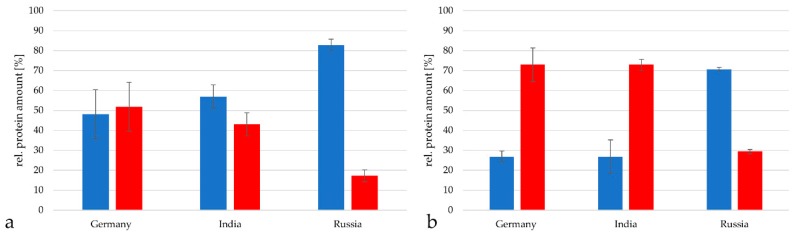
Composition of *C. tetani* proteins in the vaccines from Germany, India and Russia with tetanus neurotoxin (TeNT) in red and the remaining proteins in blue. (**a**) Analysis by the TPA-based method using the area under the peak. Only proteins with peptides in in a range from 2 × 10^7^ to 10^11^ were considered for calculation. (**b**) Analysis with the Proteome discoverer 2.2 program package.

As next step, the relative amount of tetanus neurotoxin (TeNT) in the tetanus toxoid preparations was determined. TeNT represented 48.2 ± 12.2% in the German vaccine, 43.7 ± 5.8% in the vaccine from India and only 17.2 ± 3.0% in the vaccine from Russia when analyzed with the TPA-based method (Figure 5a). When the Proteome Discoverer software package 2.2 was used, TeNT reached 72.9 ± 8.4% in the German vaccine, 72.9 ± 2.8% in the Indian vaccine and 29.4 ± 1.1% in the vaccine from Russia when analyzed with the TPA-based method (Figure 5b).

Taken together, with this method higher relative amounts of TeNT were detected; however, in all cases the Russian vaccine contained the lowest amount of TeNT. Since the toxoid was adjusted in form of flocculation units [13,14], this meant that the Russian toxoid was the most immunologically active in this comparison.

**Figure 6:**
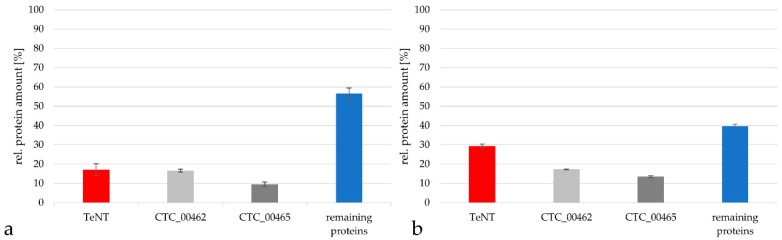
Comparison of the relative protein amount of TeNT (red), the two most prominent proteins beside TeNT (grey) and of the remaining proteins (blue) from *C. tetani* in the Russian vaccine. (**a**) Analysis by the TPA-based method using the area under the peak. Only proteins with peptides in in a range from 2 × 10^7^ to 10^11^ were considered for calculation. (**b**) Analysis with the Proteome Discoverer 2.2 program package.

When the protein content from *C. tetani* in the Russian vaccine was analyzed in more detail using the TPA-based method, the TeNT represented only 17.2 ± 3.0% of all *C. tetani* proteins, while the putative S-layer protein/N-acetylmuramoyl-L-alanine amidase CTC_00462 reached 16.7 ± 0.8% of the protein content, the putative S-layer protein CTC_00465 9.6 ± 1.1% and the remaining proteins summed up to 56.5 ± 3.0% (Figure 6a). Also in this case, the general tendency of protein distribution was similar when the Proteome Discoverer software package was used. In this case the TeNT reached 29.4 ± 1.1% of all *C. tetani* proteins, while the putative S (surface)-layer protein/N-acetylmuramoyl-L-alanine amidase CTC_00462 reached 17.3 ± 0.3%, the putative S-layer protein CTC_00465 13.5 ± 0.4% and the remaining proteins 39.8 ± 1.1% (Figure 6b).

## 4. Discussion

Proteomic data are scarce for *C. tetani* and only a few proteins have been identified in proteome studies up to now [29]. In frame of this study, we were able to identify 991 different proteins of *C. tetani* as components of tetanus toxoid vaccines. In addition, we found a highly variable number of proteins depending on the studied vaccines, which may indicate that different companies use different protocols for tetanus toxoid production.

Independent of the number of detected proteins and the protein quantification method used, the main component of all vaccines was tetanus neurotoxin. The high number of predicted cytoplasmic proteins found in this study, 64% of the total identified proteins, may be explained by cell lysis of bacteria, which leads to a release and accumulation of especially highly abundant and stable proteins such as components of glycolysis, pentose phosphate pathway, tricarboxylic acids cycle and protein turnover into the supernatant. Alternatively, nonclassical transporters or nonspecific leakage or release by exosomes was discussed for these group of proteins [5]. Shearing forces may be another source of membrane-associated proteins, resulting for example from stirring during cultivation.

From the 206 proteins present in all samples, 54 showed immunogenic potential. Especially antibodies directed against surface-exposed proteins such ABC (ATP-binding cassette) transporter components (CTC_02340, CTC_01379, CTC_00907, CTC_00860), putative S-layer proteins (CTC_02093, CTC_00691, CTC_00465, CTC_00462), flagellar components (CTC_01724) and putative adhesins (CTC_00777, CTC_00774, CTC_00771, CTC_00770, CTC_00769, CTC_00749, CTC_00747) may have an influence on host colonization by the pathogen. This may also be true, when antibodies against putative virulence factors such as proteases are induced (CTC_02507, CTC_01225, CTC_00882, CTC_00612, CTC_00519). In addition, enolase (CTC_00382) was found among the 206 proteins observed in all studied vaccines, was predicted to have immunogenic properties. This enzyme was identified earlier as one of a group of cross-reactive proteins from *C. perfringens*, which may provide protection against *C. tetani* [29].

In summary, besides the tetanus neurotoxin, which contributes between 14 and 76% to the total *C. tetani* protein content depending on the tetanus vaccine sample and the protein quantification method used, a high number of other *C. tetani* proteins were detected, which may contribute at least partially to a protection against *C. tetani* infection supporting the function of the vaccine against the devasting effects of the tetanus toxin indirectly.

Interestingly, *Clostridium botulinum* toxoid vaccination in Danish cows did not only reduce botulinum neurotoxin in cattle feces, but also the number of *C. botulinum* spores. This observation may support the idea that toxoid preparation may also protect against infection with pathogenic bacteria [30].

## 5. Conclusions

DT vaccines consist of about 20% *C. diphtheriae* and about 80% *C. tetani* proteins. Tetanus neurotoxin is a major protein in toxoid preparations; however, similar amounts of putative S-layer proteins are found as well. These and other proteins found may contribute to protection against *C. tetani* infection.

## Figures and Tables

**Table 1:** Vaccine samples analyzed in this study. The concentrations of the active components are shown in flocculation units (Lf) *^1^, international units (I.U.) *^2^ and binding units (BU) *^3^.

Origin	Company	Active components
Brazil	Butanan Institute	Tetanus toxoid ≥ 25 Lf/mL Diphtheria toxoid ≥ 2 Lf/mL
Bulgaria	InterVax for BB-NCIPD	Tetanus toxoid ≥ 20 Lf/mL Diphtheria toxoid ≥ 30 Lf/mL
Germany	GlaxoSmithKline (GSK)	Tetanus toxoid ≥ 20 I.U. Diphtheria toxoid ≥ 2 I.U.
India	Biological E (BE)	Tetanus toxoid ≥ 20 I.U. Diphtheria toxoid ≥ 2 I.U.
Russia	Microgen	Tetanus toxoid ≥ 5 BU/mL Diphtheria toxoid ≥ 5 Lf/mL

*1: Flocculation units (Lf) are defined as the equivalent of toxin to one unit of standard antitoxin or the amount of toxin that flocculates one unit of an international reference antitoxin [13,14]. *2: International units (I.U.) are defined as a certain weight of the international standard for antitoxin [15] and are equal to the corresponding amount of toxin. *3: Binding units (BU) correspond to the amount of tetanus toxoid to bind directly or compete with tetanus antibody [16] and, therefore, are equal to Lf or I.U.

**Table 2:** Proteins with predicted antigenic potential identified in all five analyzed vaccines. The localization was extracted from the UniProt database or predicted with Psortb v.3.0.2 (S: secreted, M: membrane-localized, C: cytoplasm and U: unknown, without predicted localization).

UniProt ID	Identifier	Annotation	Localization
P04958	CTC_p60	Tetanus toxin	S, M, C
Q890P1	CTC_02598	50S ribosomal protein L2	U
Q890T2	CTC_02553	Thioredoxin	M, C
Q890 × 6	CTC_02507	Tail-specific protease	M, C
Q890Z2	CTC_02490	ATP-dependent 6-phosphofructokinase	M, C
Q891M9	CTC_02340	Glycine betaine-binding protein	M
Q891P2	CTC_02327	V-type ATP synthase beta chain 2	M, C
Q891Q6	CTC_02312	Conserved protein	U
Q891U8	CTC_02265	UDP-glucose 6-dehydrogenase	M, C
Q892B0	CTC_02196	Hydroxyacylglutathione hydrolase	U
Q892H0	CTC_02129	Phage protein	U
Q892K3	CTC_02093	N-acetylmuramoyl-L-alanine amidase/putative S-layer protein	M
Q892P7	CTC_02047	Dihydrolipoyl dehydrogenase	M, C
Q892V6	CTC_01980	Uncharacterized protein	M
Q893B5	CTC_01913	Uncharacterized protein	M
Q893R9	CTC_01741	Pyruvate-flavodoxin oxidoreductase	U
Q893T5	CTC_01724	Flagellar hook-associated protein 1	S
Q894F4	CTC_01593	3-dehydroquinate dehydratase	M
Q894P1	CTC_01495	Conserved protein	U
Q894P7	CTC_01488	Fumarate reductase flavoprotein subunit	M
Q894Q6	CTC_01479	Uncharacterized protein	M, C
Q894Q7	CTC_01478	Putative histidine decarboxylase	U
Q895A1	CTC_01379	Periplasmic transport protein, nickel or dipeptide transport	M
Q895E4	CTC_01332	Transketolase	U
Q895G9	CTC_01305	Uncharacterized protein	U
Q895P6	CTC_01225	Serine/threonine protein kinase	M
Q895R2	CTC_01209	Uncharacterized protein	U
Q895T9	CTC_01178	NADH oxidase	M, C
Q896G9	CTC_01036	Uncharacterized protein	U
Q896I3	CTC_01021	Electron transport complex subunit G	M
Q896J5	CTC_01009	Conserved protein, putative N-acetylmuramoyl-L-alanine amidase	M
Q896U1	CTC_00907	D-ribose-binding periplasmic protein	U
Q896W4	CTC_00882	Carboxyl-terminal protease	M, C
Q896W8	CTC_00878	50S ribosomal protein L25	U
Q896Y3	CTC_00860	D-galactose-binding periplasmic protein	U
Q896Y6	CTC_00856	Uncharacterized protein	M, C
Q897C9	CTC_00811	Fumarate reductase flavoprotein subunit	M
Q897E8	CTC_00792	Conserved protein	M
Q897G1	CTC_00777	Putative surface/cell-adhesion protein	U
Q897G4	CTC_00774	Putative surface/cell-adhesion protein	M
Q897G6	CTC_00771	Putative surface/cell-adhesion protein	M
Q897G7	CTC_00770	Putative surface/cell-adhesion protein, big2 domain	U
Q897G8	CTC_00769	Putative surface/cell-adhesion protein	M, C
Q897I6	CTC_00749	Putative surface/cell-adhesion protein, multiple big2 domain	M
Q897I8	CTC_00747	Putative surface/cell-adhesion protein, multiple big2 domain	M
Q897P1	CTC_00691	Putative S-layer protein/internalin A-like/N-acetylmuramoyl-L-alanine amidase	M
Q897Q0	CTC_00681	Conserved protein	M
Q897W0	CTC_00612	Serine protease	S
Q898E1	CTC_00519	Zink-carboxypeptidase	U
Q898I5	CTC_00465	Putative S-layer protein	M
Q898I7	CTC_00462	Putative S-layer protein/N-acetylmuramoyl-L-alanine amidase	M
Q898R0	CTC_00382	Enolase	S
Q898S3	CTC_00369	Membrane lipoprotein tmpC	M
Q899E7	CTC_00234	Putative cell wall hydrolase	S

## Supplementary Materials

The following are available online at https://www.mdpi.com/2227-7382/7/2/15/s1, Table S1: *C. tetani* proteins identified in vaccine samples analyzed. Common proteins shaded in grey.
